# Genome-wide genetic diversity and population structure of *Garcinia kola* (Heckel) in Benin using DArT-Seq technology

**DOI:** 10.1371/journal.pone.0238984

**Published:** 2020-09-23

**Authors:** Colombe Dadjo, Aggrey B. Nyende, Nasser Yao, Ngeno Kiplangat, Achille E. Assogbadjo

**Affiliations:** 1 Institute of Basic Sciences, Technology and Innovation, Pan African University, Nairobi, Kenya; 2 Laboratory of Applied Ecology, Faculty Agronomic Sciences, University of Abomey-Calavi, Cotonou, Rep. Benin; 3 Institute of Biotechnology Research, Jomo Kenyatta University of Agriculture and Technology, Nairobi, Kenya; 4 Bioscience Eastern and Central Africa, International Livestock Research Institute, Nairobi, Kenya; 5 Animal Breeding and Genomics Group, Department of Animal Science, Egerton University, Egerton, Kenya; University of Iceland, ICELAND

## Abstract

*Garcinia kola* (Heckel) is a versatile tree indigenous to West and Central Africa. All parts of the tree have value in traditional medicine. Natural populations of the species have declined over the years due to overexploitation. Assessment of genetic diversity and population structure of *G*. *kola* is important for its management and conservation. The present study investigates the genetic diversity and population structure *of G*. *kola* populations in Benin using ultra-high-throughput diversity array technology (DArT) single nucleotide polymorphism (SNP) markers. From the 102 accessions sampled, two were excluded from the final dataset owing to poor genotyping coverage. A total of 43,736 SNPs were reported, of which 12,585 were used for analyses after screening with quality control parameters including Minor allele frequency (≥ 0.05), call rate (≥ 80%), reproducibility (≥ 95%), and polymorphic information content (≥ 1%). Analysis revealed low genetic diversity with expected heterozygosity per population ranging from 0.196 to 0.228. Pairwise F-statistics (F_ST_) revealed low levels of genetic differentiation between populations while an Analysis of molecular variance (AMOVA) indicated that the majority of variation (97.86%) was within populations. Population structure analysis through clustering and discriminant analysis on principal component revealed two admixed clusters, implying little genetic structure. However, the model-based maximum likelihood in Admixture indicated only one genetic cluster. The present study indicated low genetic diversity of *G*. *kola*, and interventions are needed to be tailored towards its conservation.

## 1. Introduction

*Garcinia kola* (Heckel) is endemic in coastal areas, lowland plains and humid lowland rainforest of West and Central Africa [1]. The tree can naturally grow up to 30 m in height with a maximum of 100 cm in trunk diameter. The species is dioecious and flowering usually occurs once per year [1]. *Garcinia kola* is an indigenous tree with cultural and medicinal importance to local populations [2]. In folkloric medicine, it has important uses in the treatment of various ailments such as cough, chest pain, tooth ache, rheumatism, hernia, dysentery and malaria [3, 4]. It is also used to induce labor for easy delivery and during socio-cultural engagement like naming and wedding ceremonies [3, 4]. However, the species is only found in the Southern areas [1] where the highest destructive anthropogenic activities are recorded. It is potentially threatened by indiscriminate bark sampling used in the preparation of different infusions [2]. These anthropogenic activities may have led to the drastic decline in natural populations of the *G*. *kola*, and now the species is classified as “*extinct in the wild*” in the Benin Red List of Threatened Species [2]. In spite of the socio-economic importance of *G*. *kola* and the current conservation status of the species in Benin, there is no report on molecular genetic diversity and population structure of *G*. *kola* in Benin.

Genetic diversity is important in species conservation and is correlated to population size [5]. Therefore, a reduction of population size is likely to lead to a reduction in genetic diversity over time [6]. Molecular markers such as restriction fragment length polymorphisms (RFLPs), single sequence repeats (SSRs), and single nucleotide polymorphisms (SNPs) have been utilized in several crop species to ascertain the genetic diversity in the gene pool [7], identify quantitative trait loci (QTLs) and candidate genes conferring valuable traits [8, 9], authenticate plant identity and the parentage of hybrids [10], generate data for gene expression profiling [11], and predict the genetic potentiality/performance of individual cultivars [12]. SNP markers are the most widely used genotyping markers due to their abundance in the genome [13]. Diversity array technology (DArT) along with other high throughput sequencing techniques have been increasingly adopted as a rapid and low-cost molecular techniques for SNP discovery, genotyping, and genetic variability analysis for various plant species as they allow for the study of the genetic diversity of a large number of entities and complex genomes [14–17]. The present study investigates the current level of genetic diversity and population structure of *G*. *kola* populations in Benin using a DArT genotyping by sequencing (GBS) approach. The specific questions addressed in this study were:

- (i) What is the amount of genetic diversity and differentiation harbored within-and among populations?
- (ii) Does population structure reflect geographic locations of collection?
- (iii) Do populations form distinct genetic clusters? An analysis of genetic diversity and structure will generate information crucial to *G*. *kola* genetic improvement activities, and provide foundation for decision-making on conservation interventions.

## 2. Materials and methods

### 2.1. Plant materials

The study was carried out on private lands between June and August 2017. Leaders of the communities as well as agriculture extension workers were made aware of our presence and the purpose of the study. Samples were collected upon owners' permission.

Eight populations of *G*. *kola*, which represent the collection locality/site, were sampled in the Atlantique, Oueme and Plateau regions of Benin (Table 1). A population was defined as a group of trees separated from their nearest conspecifics by at least 1 km [18]. The minimum distance between two sites was 5 km. A minimum distance of 50 metres between accessions/individuals was used to avoid selecting closely related individuals. A total of 102 accessions were collected (1, 13 and 88 from Plateau, Atlantique and Oueme respectively) with 1 to 54 accessions/individuals collected per population (Table 1). The number of trees sampled within each locality was proportional to the number of trees within each locality.

**Table 1:** Geographical location and size of the studied *G*. *kola* individuals sampled.

Region	Locality/Population	GPS location		
		Latitude N	Longitude E	N° of accessions sampled
Atlantique	Kpomasse-Tori	6.5318	2.1933	4
	Abomey-Calavi	6.438613	2.262541	9
Oueme	Porto-Novo	6.497333	2.640761	8
	Avrankou	6.544079	2.642105	6
	Adjarra	6.50761	2.66414	54
	Dangbo	6.603947	2.540379	10
	Adjohoun	6.63896	2.520234	10
Plateau	Ketou	7.474918	2.672445	1

Tender and apparently healthy leaf tissues were collected randomly from sampled adult trees. The samples were placed in labelled envelopes and dried in silica gel until transport to the laboratory for DNA extraction.

### 2.2. DNA extraction and purification

The genomic DNA was extracted following the cetyltrimethylammonium bromide (CTAB) DNA isolation protocol with slight modifications as described by Hanaoka et al. [19]. Extracted DNA samples were stored at -20°C for further analysis.

### 2.3. DArTseq analysis

Diversity Arrays Technology (DArT) Pty Ltd, Australia (http://www.diversityarrays.com) libraries were prepared using the *PstI-SphI* complexity reduction method [20, 21]. Around 150 to 200 ng of genomic DNA were processed in digestion/ligation reactions with *PstI* Restriction Enzyme (RE) (as a rare/primary cutter) and *SphI* RE frequent cutter at 37°C for 3 hours, then at 60°C for 20 min [20]. The forward adapter (*PstI*-compatible) included an Illumina flow cell attachment sequence, sequencing primer sequence and, varying length barcode region [20, 22] and the reverse adapter contained a flow cell attachment region and a *SphI*-compatible overhang sequence. Samples were processed in batches of 94. For each sample, amplification of the fragments containing both *PstI* and *SphI* (mixed fragments) [22] was performed using specially designed primers. PCR protocol were performed under the following conditions: initial denaturation at 94 °C for 1min followed by 30 cycles of denaturation at 94 °C for 20 sec, annealing at 58 °C for 30 sec and extension at 72 °C for 45 sec. This was followed by a final extension step at 72 °C for 7 min, and a hold at 10°C. PCR products were cleaned and a small sample (5 μl) from each PCR product was loaded on 1.2% agarose gel for quality control. A pool of all the amplified products was then assembled. Samples which did not appear to have undergone complete digestion and/or amplification were removed from downstream library preparation. Samples were normalized and pooled using an automated liquid handler, at equimolar ratios and sequencing was performed using an Illumina HiSeq 2500 platform. After cluster generation and amplification, 77 bp single-end sequencing was performed. The sequence data was analyzed using DArT analytical pipelines. In the primary pipeline, the FASTQ files were processed to filter out poor-quality sequences, applying more stringent selection criteria to the barcode region than the rest of the sequence. In that way, the assignments of the sequences to specific samples carried in the ‘barcode split’ step are very reliable. Approximately 2,500,000 (±7%) sequences per barcode/sample are used in marker calling in a high-density array. Finally, identical sequences were collapsed into ‘fastqcall files. In summary, the primary pipeline filters poor-quality sequences while simultaneously applying more stringent selection criteria to the barcode region, ensuring the reliable assignment of sequences to specific samples, and then collapses identical sequences into ‘fastqcall’ files. These are used in the secondary pipeline for DArT P/L's proprietary SNP calling algorithms (DArTsoft14). Markers were scored as binary data: ‘1’ for presence, and ‘0’ for absence and ‘-’ for failure to score. Two technical replicates of each DNA sample were genotyped to calculate the reproducibility of the marker data.

### 2.4. Quality analysis of marker data

Using the KDCompute 1.5.2 [23], each locus was then assessed for polymorphic information content (PIC), average SNP count, read depth, homozygosity, heterozygosity, call rate, frequency and reproducibility before final genotype scores were supplied by DArT Pty Ltd, Australia. Two accessions from the Adjarra population were excluded from the final dataset due to poor genotyping coverage. This was because of severe allelic dropout during the genotyping by sequencing process, which led to considerable missing genetic information. Final filtering of the SNP dataset using minor allele frequency (MAF ≥ 0.05), call rate (≥ 80%), polymorphism information content (≥ 1%) and reproducibility (≥ 95%) was done.

### 2.5. Genetic diversity and population differentiation

Genetic diversity parameters such as the average observed heterozygosity (Ho), expected heterozygosity (He), inbreeding coefficient (F_IS_) and pairwise F-statistics (F_ST)_ matrices [24] were estimated using KDCompute 1.5.2 [23]. To assess hierarchical levels of population structuring, an analysis of molecular variance (AMOVA) was computed among populations and between regions based on 10,000 permutations using Arlequin 3.5.2.2 software [25]. To avoid bias and for the purpose of software requirements, the population of one individual (Ketou) was merged to the closest population (Adjohoun). A hierarchical clustering analysis was performed based on the dissimilarity matrix, and a dendogram was generated in DARwin software 6.0.18 [26]. Discriminant analysis of principal components (DAPC) was performed using the optimum number of principal components (PCs) calculated using the α-score function in the adegenet package in R [27]. Finally, a maximum likelihood estimation of individual ancestries with no prior population assumptions was performed to assess population structure using Admixture software 1.3 [28].

## 3. Results

### 3.1. Initial marker analyses and genetic diversity

**Fig 1:**
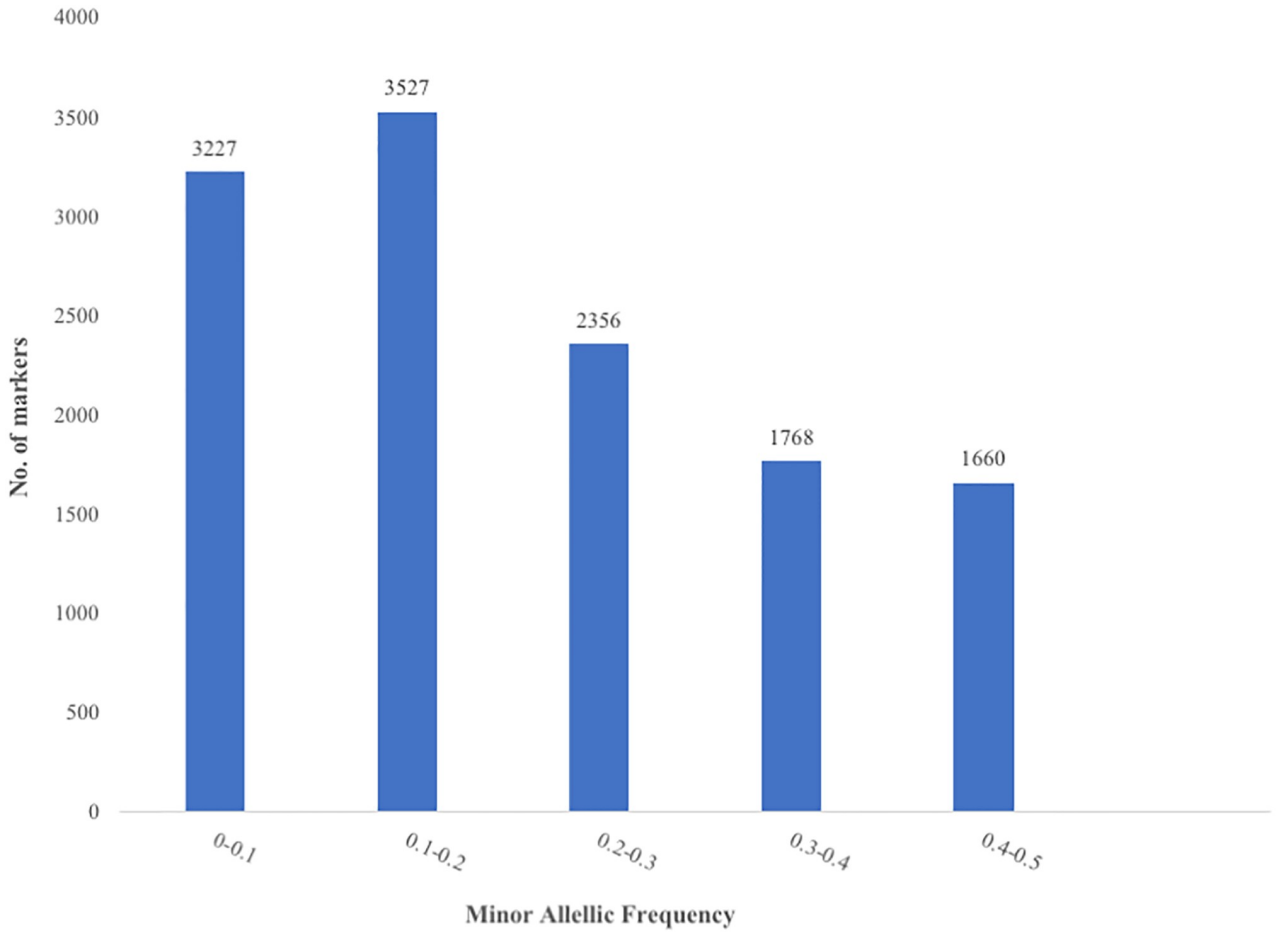
Distribution of the minor allele frequency (MAF) in 12,585 DArT-derived SNPs typed across 100 *G*. *kola* accessions; Number of SNP markers (Y axis) found within each category of MAF (X axis).

**Fig 2:**
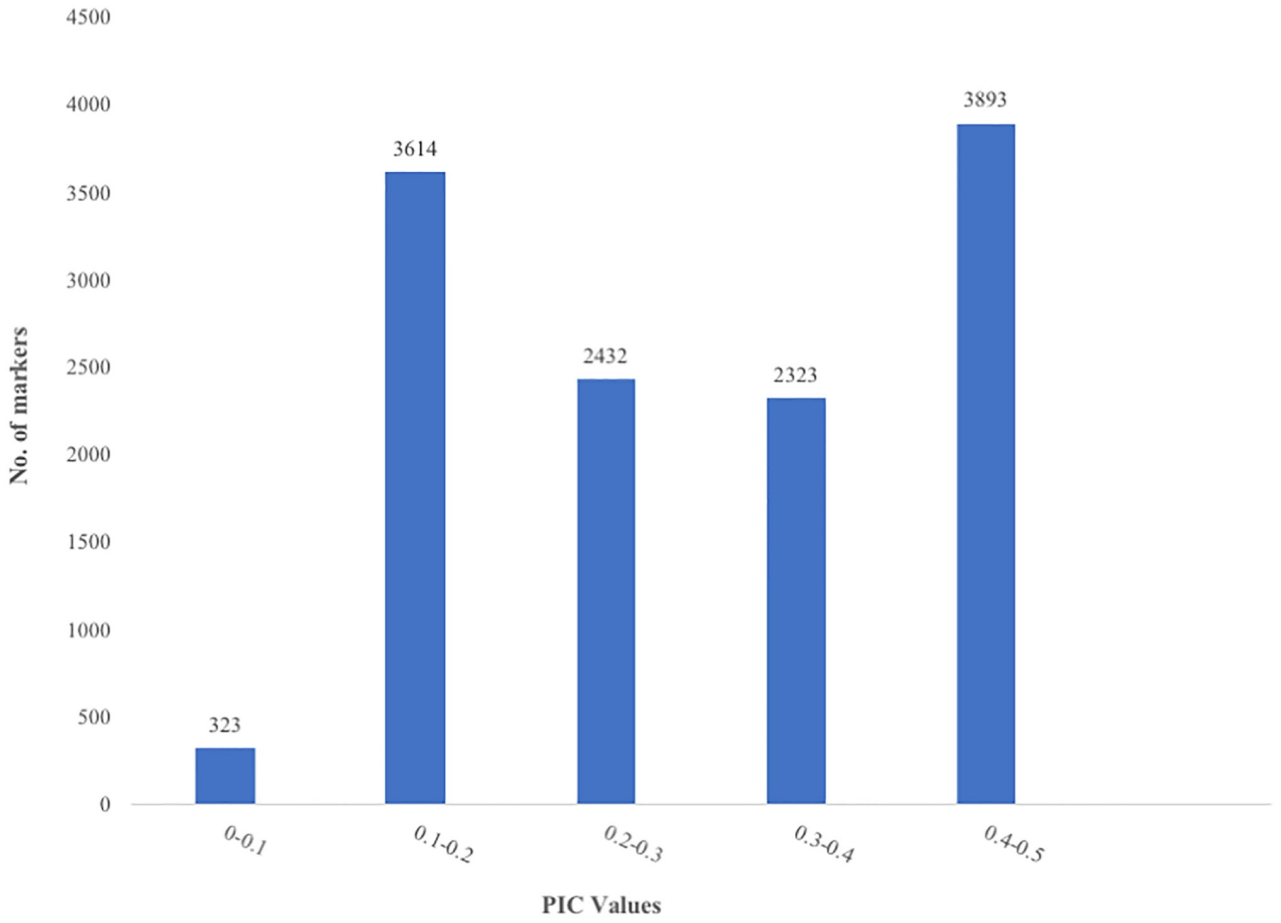
Distribution of polymorphism information content (PIC) in 12,585 DArT-derived SNPs typed across 100 *G*. *kola* accessions; Number of SNP markers (Y axis) found within each category of PIC values (X axis).

**Fig 3:**
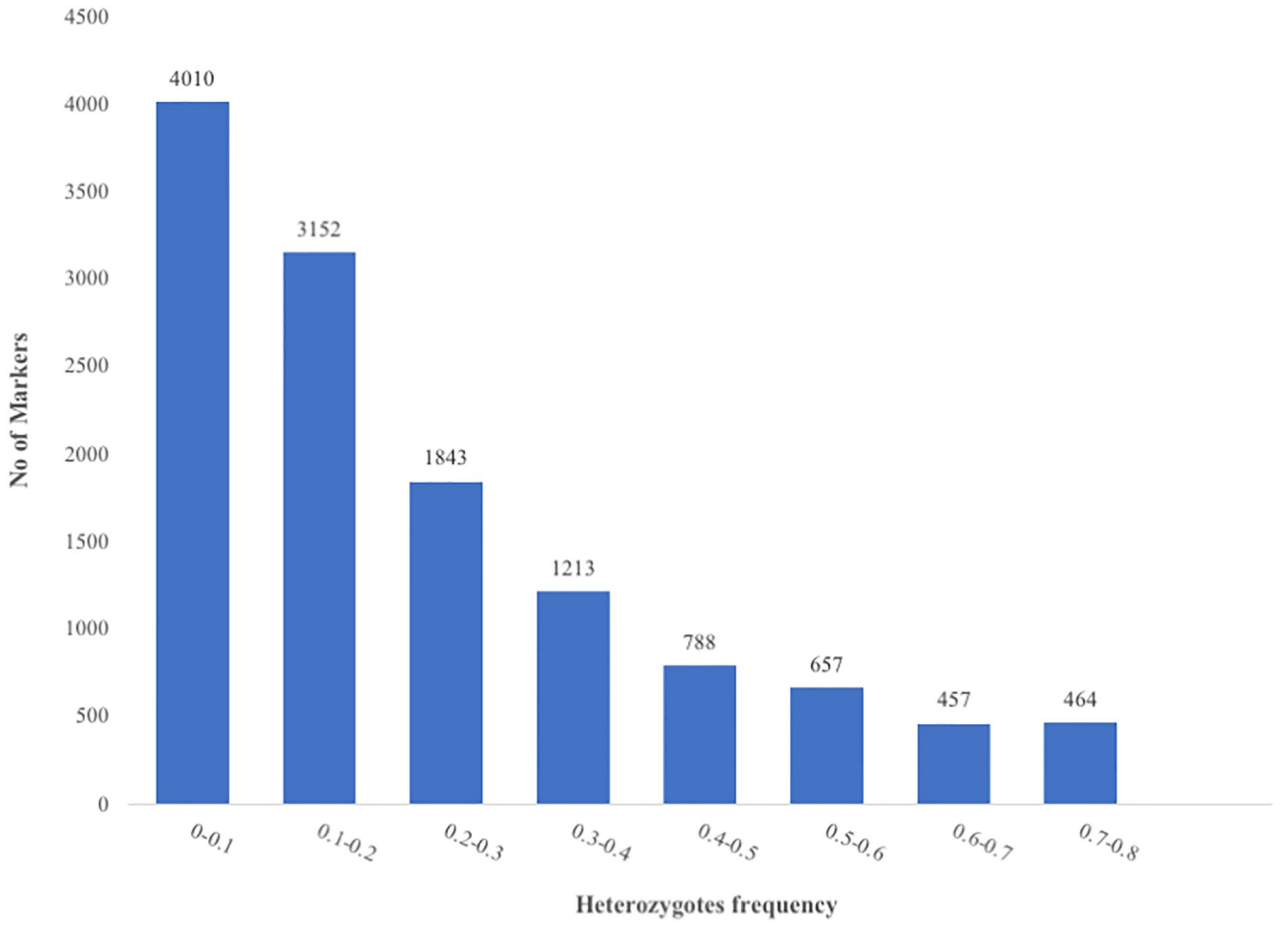
Distribution of heterozygosity in 12,585 DArT-derived SNPs typed across 100 *G*. *kola* accessions; Number of SNP markers (Y axis) found within each category of heterozygosity frequency (X axis).

The raw dataset contained a total of 43,736 SNPs genotyped across 100 accessions. After filtering the SNP dataset by minor allele frequency (MAF ≥ 0.05), call rate (≥ 80%), polymorphism information content (PIC ≥ 1%) and reproducibility (≥ 95%), 12,585 SNPs were retained (S1 Table). A total of 6,801 (54.04%) SNPs had a MAF of less than 0.2 (Fig 1). The PIC values of the markers varied from 0.095 to 0.5 (Fig 2) with an average of 0.3. About 2.57% of the markers had a PIC lower than 0.1 while 28.72%, 19.32% and 18.46% of the markers had PIC values between 0.1–0.2, 0.2–0.3 and 0.3–0.4 respectively. Informative markers with the highest PIC values (0.4–0.5) represent 31% of the markers used. The proportion of heterozygosity ranged from 0 (1 SNP) to 0.8 (7 SNPs) with an average of 0.23 (Fig 3). However, more than half of the markers (56.92%) had a proportion of heterozygosity lower than 0.2 across the accessions.

Analysis of diversity revealed low diversity with He values per population ranging from 0.196 to 0.228 while Ho values per population ranged from 0.223 to 0.248. The highest He value was observed in the Adjarra population and the lowest in the Kpomasse-Tori population (Table 2). The F_IS_, which measures the inbreeding of an individual within a sub-population was significantly high (ranging from 0.781 to 0.848) across all the populations (Table 2).

**Table 2:** Genetic diversity parameters of *G*. *kola* populations.

Populations	He	Ho	F_IS_
Kpomasse-Tori	0.196	0.227	0.781
Abomey-Calavi	0.212	0.229	0.824
Porto-Novo	0.207	0.230	0.829
Avrankou	0.216	0.248	0.847
Adjarra	0.228	0.233	0.848
Dangbo	0.214	0.223	0.823
Adjohoun	0.221	0.240	0.827

He, Expected heterozygosity; Ho, Observed heterozygosity; F_IS_, Inbreeding coefficient

### 3.2. Population relatedness and structure

In order to test for the subdivision of the Garcinia populations, an AMOVA and a pairwise genetic differentiation estimates (F_ST_) were performed for the 12,585 retained SNP markers. The results of the AMOVA (Table 3) indicated significant but weak genetic structure between regions and among populations. Only 0.86% of the genetic variation was partitioned among regions (P < 0.001) and 1.28% among populations (P < 0.001). Most of the variations (97.86%) were observed among the individuals within populations (P < 0.001).

**Table 3:** Analysis of molecular variance among *G*. *kola* populations in different regions in Benin.

Source of variation	Sum of squares	Variance components	Percentage variation	P-value
Among regions	2193.22	12.019	0.86	< 0.001
Among populations within regions	8446.83	17.782	1.28	< 0.001
Within populations	230095.38	1362.447	97.86	< 0.001
Total	240735.43	1392.250	100.00	

Pairwise genetic differentiation estimates (F_ST_) also indicated significant (P <0.001) but low levels of genetic differentiation (Table 4). The highest genetic differentiation were found between the Kpomasse-Tori and Porto-Novo (F_ST_ = 0.082) populations.

**Table 4:** Pairwise genetic differentiation estimates (F_ST_) matrix for *G*. *kola* populations.

Populations	Kpomasse-Tori	Abomey-Calavi	Porto-Novo	Avrankou	Adjarra	Dangbo
Abomey-Calavi	0.016	-				
Porto-Novo	0.082	0.046	-			
Avrankou	0.043	0.013	0.033	-		
Adjarra	0.044	0.016	0.034	-0.001	-	
Dangbo	0.046	0.018	0.035	0.002	0.003	-
Adjohoun	0.07	0.035	0.051	0.013	0.023	0.020

**Fig 4:**
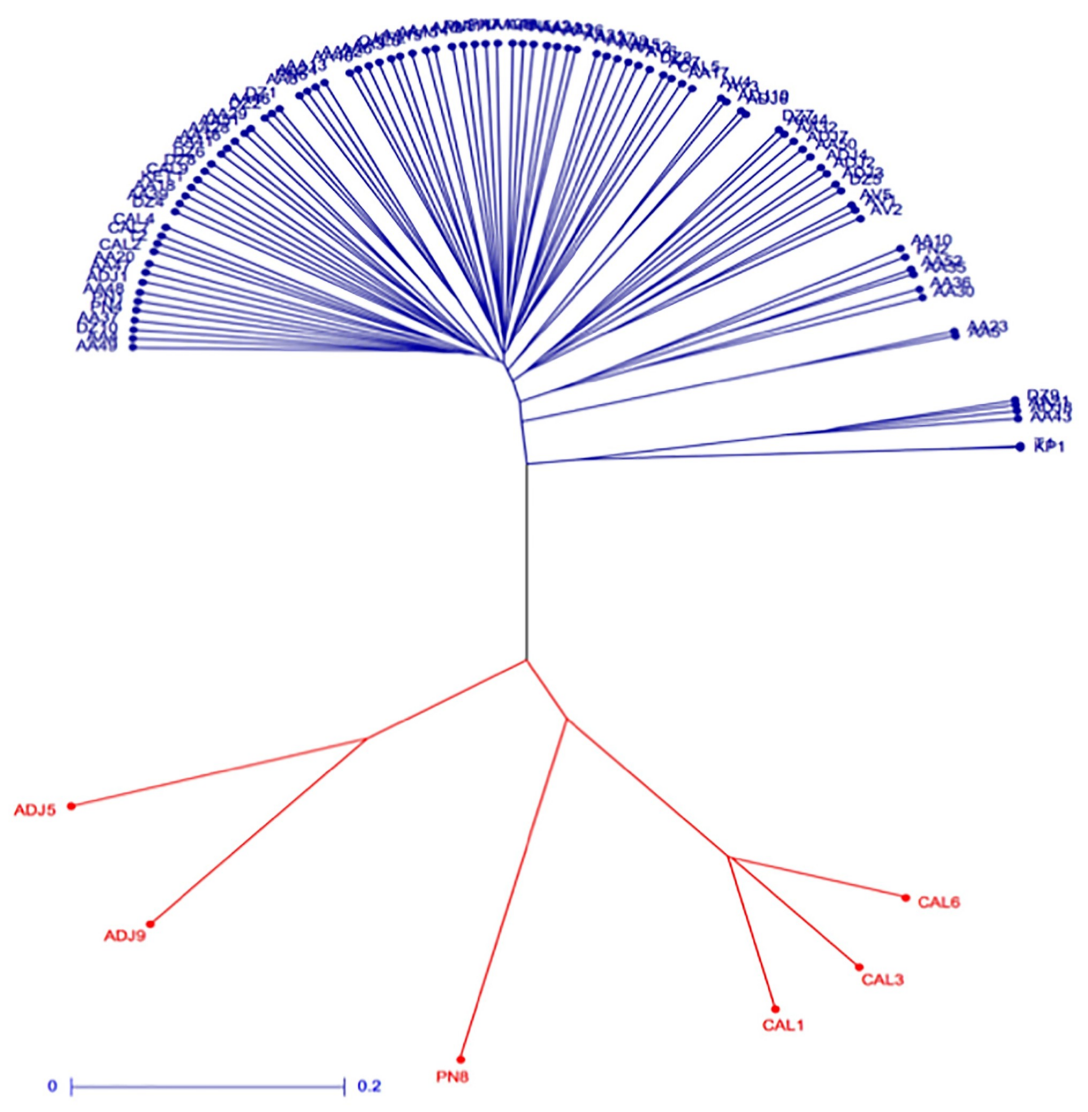
UPGMA dendogram of *G*. *kola* accessions based on dissimilarity matrix between pair of accessions. Blue: Cluster I; Red: Cluster II.

The unweighted pair group method of arithmetic averages (UPGMA) analysis clustered the 100 *G*. *kola* accessions into two admixed clusters (Fig 4). Cluster I was composed of 94 accessions from all sampled populations while cluster II was composed of 6 accessions. The result showed no clustering based on geographical locations (Fig 4).

**Fig 5:**
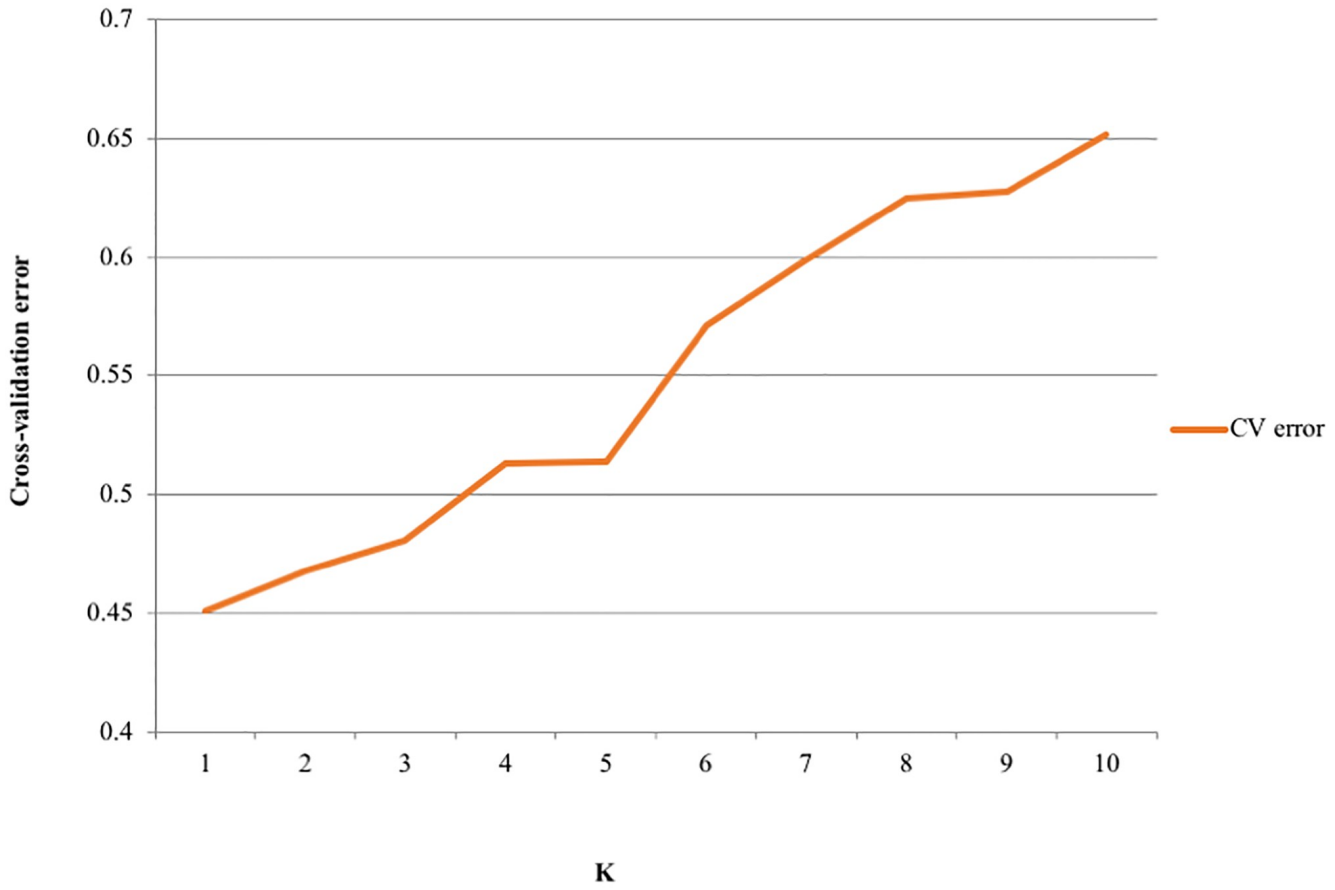
Cross-Validation error (CV) plot for K = 1 to K = 10 in *G*. *kola* accessions using Admixture.

The visualization of the population structure using DAPC displayed two admixed genetic clusters (S1 Fig). This genetic structure is similar to the high admixture observed through the UPGMA tree, indicating high genetic relatedness among the accessions. However, the admixture analysis detected the lowest cross-validation error at K = 1 (Fig 5). Indeed, the best value of k clusters is the value with the lowest cross-validation error.

## 4. Discussion

Assessment of genetic diversity and population structure of *G*. *kola* in Benin is important for the management and conservation of the species. This study provides the first-ever molecular assessment of *G*. *kola* diversity and population structure through a genome-wide SNP dataset. In this study, 12,585 informative DArTseq SNP markers were identified across 100 *G*. *kola* accessions. The PIC values were useful in studying the level of polymorphisms among the accessions. The average PIC of 0.3 indicates the markers to be moderately informative. The average PIC is also close to the value obtained for SNP markers identified using GBS in the study of rice [29] and wheat [30] diversity. A previous study on *G*. *kola* in Nigeria using RAPD markers reported a PIC value of 0.93 [31]. The bi-allelic nature of DArT-SNP markers for which the maximum value for PIC is 0.5, compared to multi-allelic RAPD markers with the maximum PIC value of 1 [32] can explain the difference in PIC values observed in the two marker systems. Low to moderate levels of observed heterozygosity (0.223–0.248) in the analyzed populations in this study point to high heterozygote deficiency. In contrast, very high heterozygosity was observed in *G*. *kola* accessions from Nigeria [31]. The low heterozygosity may be due to a severe bottleneck event that occurred during domestication and selection [33]. In Benin, *G*. *kola* is only found in home gardens, farms, and fallows and therefore may have been subjected to voluntary or involuntary selection. The results of this study are consistent with other studies reporting a reduction in genetic diversity in domesticated crops as compared to their wild progenitors [29, 34]. Heterozygote deficit has been observed in many threatened species such as *Cycad balansae* [35], *Glyptostrobus pensilis* [36], *Pulsatilla patens* [18]. Furthermore, the results of this study also reveal very high levels of inbreeding (F_IS_ = 0.781–0.848). This could be attributed to self-pollination in *G*. *kola* populations [18]. Indeed, *G*. *kola* is a dioecious species and the ability of *G*. *kola* to mate with half sibs may have resulted in inbreeding among closely related individuals. Low genetic diversity and inbreeding depression have been observed in many studies on threatened species as a consequence of a decrease in population size. [18, 37]. This is likely the case in the studied populations of *G*. *kola* in Benin, which confirms the recent report of the species' disappearance in the wild [2] with a limited number of accessions found in some populations.

Population differentiation is important for understanding the relative effect of evolutionary gene flow, mating system, selection, adaptation, and genetic drift on populations [38]. Pairwise genetic differentiation estimates (F_ST_) values is a measure of population substructure and is useful in examining the overall genetic differentiation/divergence among populations. The F_ST_ values below 0.05 indicate low genetic differentiation, while values between 0.05–0.15, 0.15–0.25, and above 0.25 indicate moderate, high, and very high genetic differentiation respectively [39]. In the present study, pairwise F_ST_ showed low but significant (p < 0.05) differentiation among the studied populations. It was also observed that genetic variation was mainly found within populations (97.86%). This could be due to the small distribution range of *G*. *kola* in Benin and the short distances between the studied populations, which facilitate gene flow between populations. In addition, *G*. *kola* populations in Benin are under heavy anthropogenic exploitation [2], and human activities significantly affect the dynamics of genetic differentiation [40]. Discriminant analysis of principal components (DAPC) and the UPGMA analyses partitioned the 100 *G*. *kola* individuals into two principal genetic clusters. Hierarchical clustering analysis performed on *G*. *kola* accessions in Nigeria also revealed two clusters [31]. However, in the present study, an admixture of almost all the populations was noted within the two clusters. The presence of admixture within the two genetic clusters implied the lack of any discernable population structure [41], thus further indicating that interbreeding or sharing of alleles has occurred between the populations. An admixture analysis was performed with the program admixture, which reduces the false-positive rates, corrects for bias toward spurious admixture, and allows identification of different mating systems in structured as well as unstructured populations [42]. A finding of K = 1 suggests the accessions in this study are actually part of the large, non-contiguous, single population with low genetic differentiation and high gene flow.

## 5. Conclusion and recommendations for future research

The present study provides insights into the molecular diversity of *G*. *kola* in Benin. Results indicate low genetic variation among the studied populations and high variation among individuals within populations. Finally, population structure analysis indicates the studied populations of *G*. *kola* to be a single population. However, it is not possible to ascertain if these results and observations can be extrapolated to the larger distribution range of *G*. *kola* because of the small distribution of the species in Benin. Sampling from a larger area, within the natural range of *G*. *kola* in West Africa, would provide a much more robust representation of genetic diversity and population structure of the species throughout its native range.

## Supporting information

S1 Table List of SNP markers used. (XLSX)

S1 Fig A plot of the discriminant analysis of principal components (DAPC) against the discriminant function retained, indicating the presence of two genetic clusters of *G*. *kola*. The plot was generated using the most informative 40 PCs identified from all the 12,585 SNPs dataset across 100 *G*. *kola* accessions in the R package adegenet.
(TIF)
