# Supplementary figures and images for: Genome-wide genetic diversity and population structure of Garcinia kola (Heckel) in Benin using DArT-Seq technology
Source: PLoS One. 2020 Sep 23;15(9):e0238984. doi: 10.1371/journal.pone.0238984 (PMC7511007; doi:10.1371/journal.pone.0238984)

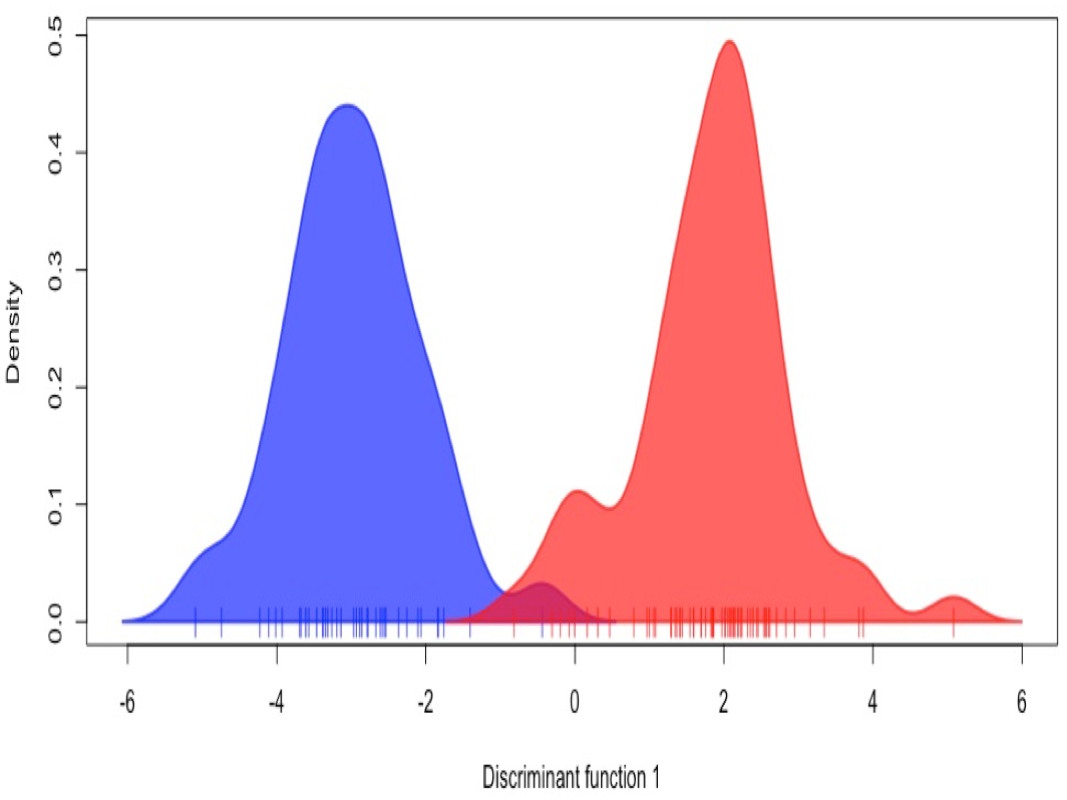

Supplement: S1 Fig — The plot was generated using the most informative 40 PCs identified from all the 12,585 SNPs dataset across 100 G. kola accessions in the R package adegenet. (TIF) [file pone.0238984.s002.tif]
